# Pacemaking Activity in the Peripheral Nervous System: Physiology and Roles of Hyperpolarization Activated and Cyclic Nucleotide-Gated Channels in Neuropathic Pain

**DOI:** 10.7759/cureus.11111

**Published:** 2020-10-23

**Authors:** Fan Liu, George Y Wuni, Ronak Bahuva, Muhammad Ahsan Shafiq, Boula S Gattas, Crystal N Ibetoh, Eugeniu Stratulat, Domonick K Gordon

**Affiliations:** 1 Internal Medicine, California Institute of Behavioral Neurosciences & Psychology, Fairfield, USA; 2 Internal Medicine, University at Buffalo, Buffalo, USA; 3 Internal Medicine, Rawalpindi Medical University, Islamabad, PAK; 4 Cardiology, Metropolitan Cardiovascular Consultants, Beltsville, USA; 5 Neuroscience, California Institute of Behavioral Neurosciences & Psychology, Fairfield, USA; 6 Obstetrics and Gynecology, California Institute of Behavioral Neurosciences & Psychology, Fairfield, USA; 7 Internal Medicine, Scarborough General Hospital, Scarborough, TTO

**Keywords:** ih, hyperpolarization-activated cyclic nucleotide gated channels (hcn), pge2, neuropathy, hcn blocker, inflammatory pain, neuropathic pain, camp, ivabradine, ectopic discharges

## Abstract

The most famous pacemaking activity found in the human body is in the cardiac system. However, pacemaking is also widely present in the nervous system. The ion channels responsible for the pacemaking activity are called hyperpolarization-activated and cyclic nucleotide-gated (HCN) channels. HCN channels are activated during hyperpolarization and create an inward current named I_h_ containing mixed sodium and potassium ions. The molecular mechanism of these unique features remains mysterious. In the peripheral nervous system (PNS), pacemaking is unique because it is only present in pathologic states when nerve damage occurs and leads to neuropathic pain. For this reason, pacemaking in neuropathic pain is also known as ectopic discharge. In our literature review, the HCN channel physiology is one of the research interests. We will present studies exploring the molecular mechanisms involved in HCN gating and ion permeability. The second research question is, what makes the pacemaking activity unique in the PNS? Thus, our paper will include studies that discuss the role of HCN channels in neuropathic pain. Given the fundamental role of HCN channels in regulating neuronal cells' discharge activity, the modulation of their function for therapeutic purposes could be useful in various pathological conditions. Here we review the present knowledge of the efficacy of HCN blocker treating neuropathic pain in humans.

## Introduction and background

As a fundamental physiological process, pacemaking is one of the most attractive topics for investigators. The realization of pacemaking in the human body initially discovered when seeing the heart was beating by itself after being isolated from the body. Such a phenomenon was documented as early as in the second century AD by Claudius Galen. He wrote, "the heart, removed from the thorax, can be seen to move for a considerable time … a definite indication that it does not need the nerves to perform its function." [1].

For the next few centuries, the pacemaking process remained mysterious. Thanks to the development of electrophysiological techniques in the late 19th to early 20th century, Noma and Irisawa were the first research group that discovered a unique current [2]. This current was quite "funny" or "queer" because it was an inward current, and the activation occurred during hyperpolarization [3].

In the late 20th century, different research groups collectively discovered the ion channels responsible for this "funny" current. In their molecular studies, the ion channel was a cyclic nucleotide-gated channel with proved to have pacemaking characteristics. Additionally, the mammalian sinoatrial node (SAN) pacemaking channels belong to this novel channel family. Because of these discoveries, the new ion channels were named hyperpolarization-activated and cyclic nucleotide-gated (HCN) channels [4,5,6,7]. HCN channels belong to the voltage-gated potassium (Kv) channel family based on its amino acid sequence [6]. Despite being in the same family, HCN channels behave quite differently compared to other Kv channels. For example, HCN channels are activated by hyperpolarization, whereas other Kv channels are activated by depolarization. Many Kv channels are potassium specific. However, HCN has both potassium and sodium ion permeability [8]. The molecular mechanism of these unique features remains mysterious.

In mammals, there are four HCN isoforms (HCN1-HCN4). These isoforms are widely distributed in the cardiac and nervous system [9]. The current generated by HCN channels is called I_f_, I_q_, or I_h_ (I for current, f for funny, q for queer, and h for hyperpolarization-activated), which can be used interchangeably. Throughout the text, we will use I_h_ to indicate the current. I_h_ is most well known for its proposed role in the generation of spontaneous pacemaker activity in the heart and brain. Thus, I_h_ is also known as the pacemaker current. Besides controlling pacemaker activity, I_h_ also has various non-pacemaking functions such as determination and limitation of resting potential, alternation of membrane resistance, regulating dendritic integration, and synaptic transmission and participation in sensation [9].

Given the diverse function of HCN channels, pathologies of such channels link to a spectrum of diseases such as arrhythmias, epilepsies, and neuropathic pain [10]. Our review paper is particularly interested in the pacemaking process present in the peripheral nervous system (PNS), which leads to neuropathic pain. The pacemaking found in the nociceptive system is quite different from the pacemaking located in the heart and the brain. The ectopic firing of nociceptive nerves only occurs in pathological conditions. Somehow, HCN channels in the peripheral nerve are changed, by pathology, from a non-pacemaking function to a pacemaking function. We can't help but ask: what is the mechanism of HCN channels in PNS that allows them to produce ectopic firing?

To date, ivabradine is the first and the only HCN channel blocker that is used in clinical therapy. It is approved by the European Medicines Agency (EMA) for an alternative treatment option for chronic stable angina pectoris when beta-blocker is contraindicated and for the treatment of heart failure. In the United States, the Food and Drug Administration (FDA) only approved ivabradine as a treatment to reduce rehospitalization risk in chronic heart failure patients [11]. Ivabradine is an excellent bradycardic agent because it cannot cross the blood-brain barrier [12]. This property also makes ivabradine a potential analgesic for treating peripheral neuropathy. The current treatments for peripheral neuropathy are opioids, anticonvulsants, and antidepressants, which may cause unwanted side effects in the central nervous system (CNS) [13]. In comparison, HCN blockers such as ivabradine will not lead to these side effects. The analgesic effect of ivabradine has been studied extensively in animal models with promising results [14]. However, ivabradine's possible analgesic effect on treating pain in humans has not been investigated in detail. Thus, we are researching how effective ivabradine is in treating neuropathic pain in human clinical trials.

In our literature review, we will first talk about the basic properties and physiology of HCN channels. Then we will incorporate the HCN channels mechanism into its contribution to neuropathic pain. Lastly, we will review the clinical trial that tests the efficacy of ivabradine as an analgesic drug.

## Review

Method

For this traditional review, no guidelines were used while conducting our research. We performed a literature search using PubMed and Google Scholar with keywords neuropathic pain, HCN channels, HCN blocker, ivabradine, HCN gating mechanism, pacemaking in the peripheral nervous system, and central sensitization. Studies were filtered based on specific inclusion and exclusion criteria.

Each article title was individually read and used to determine the relevance to our research topic. If deemed relevant, the abstract was then reviewed, and its relevance was determined. If considered relevant, the full article was read and included in the study. All results with titles, abstracts, or full articles that were not deemed relevant based on this review process were excluded from our paper. 

The specific inclusion and exclusion criteria used in this review article are: Inclusion Criteria: a) Studies and articles published between the years 1970-2020, b) Studies focus on HCN activity in the peripheral nervous system and the spinal cord, c) Studies include clinically relevant HCN channel blockers such as ivabradine, d) Studies in English. Exclusion Criteria: a) Studies written in other languages, b) Studies focus on HCN activity in the brain.

Hyperpolarization-activated cyclic nucleotide-gated channels: basic facts and physiology

Basic Facts: Molecular Structure of HCN Channels

HCN channels are tetramers. There are four different genes encoding four isoforms (HCN1-4). These isoforms are the subunits of HCN channels that can form different homotetramers or heterotetramers with distinct biophysical properties [15]. Structurally, each subunit can be divided into three domains: a cytosolic amino (N)-terminal domain, a transmembrane domain, and a cytosolic carboxy (C)-terminal domain. 

The transmembrane domain contains six transmembrane α helices (S1-S6). The ion-conducting pore locates between four pairs of S5 and S6. C-terminal part of S6, facing the cytoplasm, forms the gate, which opens during hyperpolarization [8]. There is a pore-forming loop, called P-loop, located between each pair of S5 and S6. S5, S6, and P-loop together form the pore domain (PD) in one subunit of the HCN channel [8]. Four P-loops together constitute the selectivity filter of an HCN channel. The amino acid sequence of each P-loop can be divided into two parts. A highly conserved region called the GYG (glycine-tyrosine-glycine) motif is crucial for potassium ion selection. The rest of the P-loop has divergent amino acid sequences among HCN channels and other Kv channels [9,16]. Because HCN channels are both permeable to sodium and potassium ions, it was initially hypothesized that the non-conserved amino acids in selectivity filters account for low potassium selection in HCN channels [17]. Ion permeability will be discussed in detail in the next section.

S1-S4 forms the voltage-sensing domain (VSD) located peripherally to the pore region [8]. The S4 α helix carries nine arginines or lysines regularly spaced at every third position. Arginines or lysines make S4 positively charged and function as a voltage sensing [18]. S4 is mobile; its position varies with the action potential. More importantly, the S4 movement is conserved between Kv and HCN channels. Both types will have S4 moves upward during depolarization and downward during hyperpolarization regardless of having opposite gating mechanisms [19]. The amino acids connecting the VSD and pore domain are called the S4-S5 linker. In some studies, it is thought that the S4-S5 linker has an essential role in the HCN gating mechanism. However, the exact gating mechanism remains debatable [8,20,21]. The function of the S4-S5 linker and gating mechanism will be discussed in detail in the later section.

The C-terminal domain is following S6, which is composed of two parts: cyclic adenosine monophosphate (cAMP)-sensing domain and carboxy termini. cAMP-sensing domain can be subdivided into two functional parts: C-linker and cyclic nucleotide-binding domain (CNBD). C-linker is the connecting wire between the C-terminal domain and S6 of the transmembrane domain. CNBD consists of β-roll and C-helix; it is a 120 amino acid region responsible for cAMP-mediated regulation. CNBD is highly conserved as well. In contrast, the last part of the C-terminal domain, carboxy termini, has lots of variation among HCN isoforms [9]. After binding cyclic AMP or cyclic GMP to the channel, the CNBD-C-linker facilitates HCN channel activation [6,16,22,23].

Different HCN isoforms have a different response to cAMP. HCN1 and HCN3 are cAMP insensitive, whereas HCN2 and HCN4 are sensitive to cAMP. When increasing intracellular cAMP, cAMP-sensitive channels will shift its activation range to more positive membrane potentials. This shift will activate other voltage-dependent channels to continue to depolarize the membrane potential [22]. HCN channels are activated by direct binding of cAMP and are independent of protein kinase A (PKA) [24]. Each HCN isoform also has a different time constant (a parameter that measures how fast an ion channel activates/deactivates). HCN1 channel has the lowest time constant; thus, the fastest activation. HCN2-4 are slow activating channels compared to HCN1 [17,25]. The I_h_ found in many large somatosensory neurons is thought to be generated by HCN1 because the fast I_h_ disappears when HCN1 is genetically deleted. When HCN2 is deleted in small nociceptive neurons, most slow and cAMP-dependent I_h_ disappear, indicating that HCN2 is the main contributor [23].

Physiology: I_h_ is a Current That Contains Mixed Cations 

The HCN channels are unique compared to other relatives in the voltage-gated potassium channel family because they conduct a mixed cation current carried by sodium and potassium. Because of distinctive ion permeability, Kv and HCN channels will have different reversal potential. Since Kv channels are only permeable to potassium, the reversal potential will be -70 to -80 mV. In contrast, HCN will have reversal potential around -20 mV to -40 mV due to sodium conductance. When the HCN channels open at hyperpolarization, sodium ions will flow into the cell and lead to depolarization [5,6,7,8,9]. Surprisingly, selectivity filters of HCN channels vary similar to potassium-selective voltage-dependent channels (Kv) because they contain all the essential amino acids required for selecting potassium [8]. Despite the similarity at the molecular level, the HCN channel has a sodium/potassium permeability ratio of 1:3 to 1:5, whereas Kv has a ratio of 1:1000 [26]. 

These observations may imply some structural differences present in HCN channels contribute to the mixed cation conductance. By studying the cryo-electron microscopy structures of the human HCN1, Lee and MacKinnon found a possible explanation. When comparing the HCN1 structure and a closely related potassium-selective channel (KcsA), it is found that in two of the four selectivity filters, the tyrosine residue in the GYG motif reoriented 180 degrees. As a result, two tyrosine residues with their ion-binding carbonyl oxygen atoms are embedded inside instead of exposing them to the pore. This structural change leads to two tyrosine residues in HCN channels that cannot participate in potassium binding. Since two tyrosine residues bind to one potassium, HCN pore will only bind one potassium. Single ion binding pores allow more space, which is kinetically favorable for passing sodium [8]. 

Why are two of the tyrosine residues embedded? Lee and MacKinnon believe the non-conserved amino acids in selectivity filters are essential for the structural change [8]. On KcsA pore helix, Trp68 and Thr72 are two amino acids forming hydrogen bonds with tyrosine in the GYG motif [27,28]. In HCN, the Trp and Thr on the pore helix are replaced by Lys and His. It is believed that losing the stabilizing effect from hydrogen bonds contributes to the orientation change of tyrosine residues [8]. Lee and MacKinnon's findings coincide with the initial hypothesis provided by Robinson and Siegelbaum [17].

Physiology: Unique Gating Mechanism; HCN Channels Open at Hyperpolarization.

Most voltage-dependent ion channels open when the membrane is depolarized. HCN channels behave in the opposite manner. There are currently two models to explain voltage-gated ion channels' gating mechanism: canonical VSD-PD coupling and non-canonical VSD-PD coupling [21]. In the canonical model of voltage-dependent coupling, VSD and PD interactions are traduced by a covalent linkage, the S4-S5 linker, in a domain-swapped fashion, which allows VSD interacts with neighboring PD [20,21,29]. Movement of each S4 pulls on the S4-S5 linker, which in turn opens a single gate in the pore [29]. To domain-swap, ion channels must have long S4-S5 linkers. Many depolarization-activated Kv channels are canonical coupled [8,20,21,29]. In contrast, for ion channels with very short S4-S5 linkers, a VSD cannot interact with neighboring PD. Instead, it can only interact with the PD on the same subunit. This type of VSD-PD interaction is called non-domain-swapping. In non-domain-swapping, non-covalent interactions between amino acids side chains from VSD (S4) and PD (S5) become the dominant force for VSD-to-PD coupling, and such coupling mechanism is called non-canonical VSD-PD coupling [20,21,29]. Once again, non-canonical VSD-PD coupling is a model that is mainly formulated based on studies of depolarization-activated Kv channels that have short S4-S5 linkers [30,31]. However, it is still not clear which model is the best fit for the HCN gating mechanism.

Lee and MacKinnon's work does not stop at investigating HCN channel ion permeability. Further, they look at the cryo-electron microscopy structures of the human HCN1 channel and identify any structure that could explain the hyperpolarization gating mechanism. By comparing HCN1 and Kv10.1 channel, a channel that closely relates to the HCN channel but opens at depolarization, it is found that S4 is much longer in HCN1 because it contains two extra helical turns. This unique structure allows S4 to extend into the cytoplasm even when it stays in the upward position (depolarized conformation). Lee and MacKinnon believe that the extra length of S4 functions like a "hook" to stabilize closed gates at depolarization. During depolarization, S4 moves upward, and its cytoplasmic portion will make contact with the S4-S5 linker. Such contact will twist the C-linker to stabilize the cytoplasmic portion of the pore, S6 C-terminus, in a closed conformation. According to this model, the upward movement of the voltage sensor (S4) has an inhibitory effect on the pore opening. When the membrane potential is hyperpolarized, the voltage sensor moves downward and releases the packing arrangement between S4, S5, and S6 helices, thus enabling the pore to open [8]. 

Recently, two independent research groups, Flynn et al. and Cowgill et al., tested Lee and MacKinnon's model by truncating the S4-S5 linker or shortening S4 as much as three helical turns. Unfortunately, both studies cannot eliminate gate activation upon hyperpolarization, leading to the conclusion that the S4-S5 linker and S4 extra length are not required for hyperpolarization-dependent gating [20,29]. Nonetheless, the significance of Lee and MacKinnon's work on HCN gating activation is that they provide a possible molecular mechanism suggesting the HCN gating mechanism fits the non-canonical VSD-PD coupling model. More importantly, both studies also reach a similar conclusion that HCN gating is most likely due to non-covalent interactions between S4 and S5 [20,29]. However, which amino acids are responsible for S4 and S5 interaction remain unclear. 

**Figure 1:**
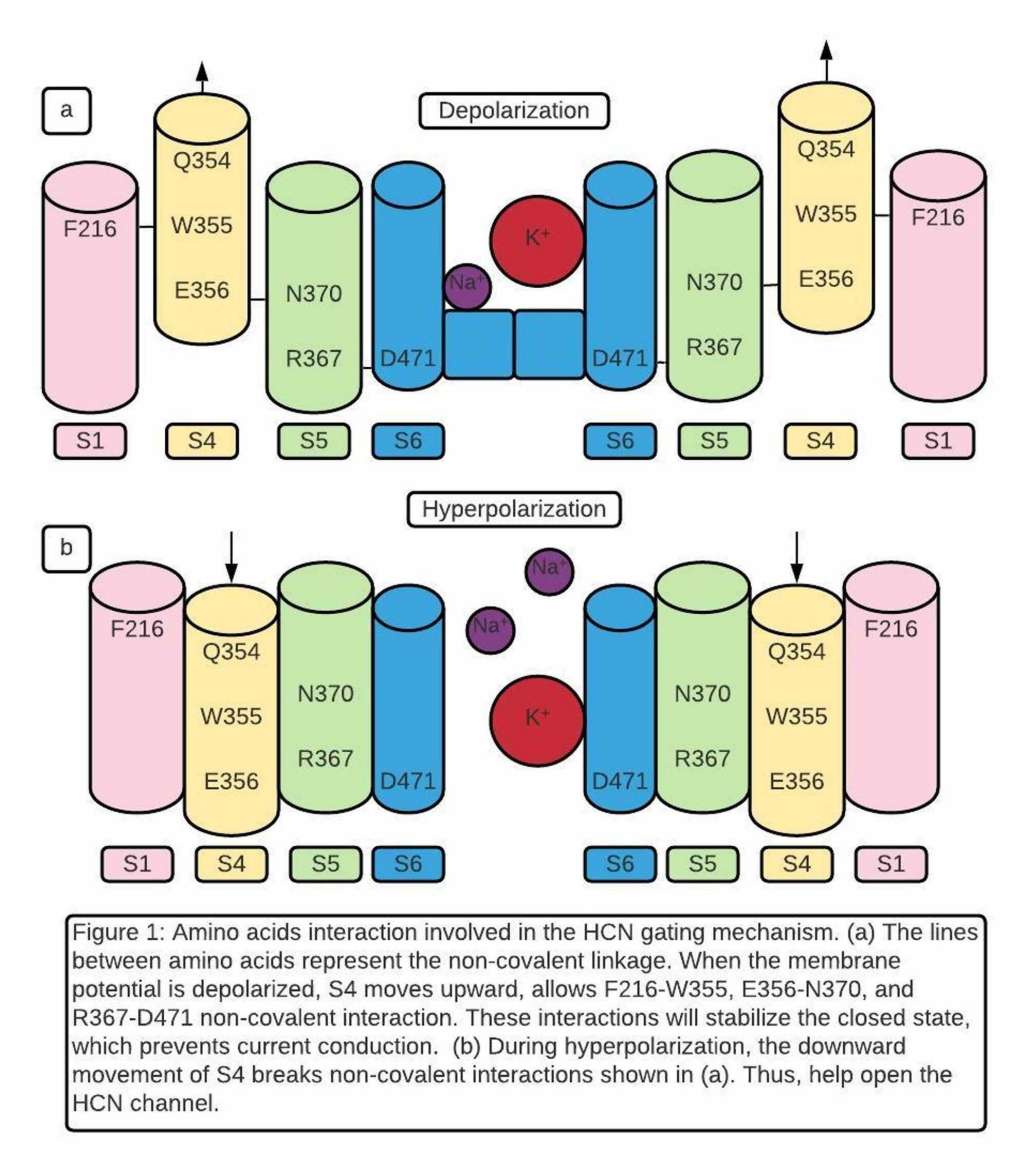
Amino acids interaction involved in the hyperpolarization-activated and cyclic nucleotide-gated (HCN) gating mechanism. Amino acid abbreviations: F (Phenylalanine); Q (Glutamine); W (Tryptophan); N (Asparagine); E (Glutamate); R (Arginine); D (Aspartate)

The most recent study by Ramentol et al. completed the final step of understanding the HCN gating mechanism. The data identified several specific amino acids that are essential to HCN gating. These amino acids are listed in Table 1. F216 comes from S1. Q354, W355, and E356 form the QWE motif in S4. N370 and R367 are located in S5. R367 is one helical turn below N370. S4 QWE motif is located between S1 and S5. When the membrane is depolarized, and S4 is up, F216 (S1) will link to W355 (S4), E356(S4) will link N370 (S5), and R367 (S5) will link to D471 (S6) (Figure 1). These interactions are non-covalent and are believed to stabilize the closed state [2,32]. 

**Table 1:** Amino acids participating in gating mechanism of hyperpolarization-activated and cyclic nucleotide-gated (HCN) channel. Amino acid abbreviations: F (Phenylalanine); A (Alanine); Q (Glutamine); W (Tryptophan); N (Asparagine); E (Glutamate); R (Arginine); D (Aspartate)

Amino Acids	Location	Mutation	Mutation Effect	Authors
F216	S1	F216A	Destabilize the closed state (12% conductance remaining at positive voltages).	Ramentol et al. [21]
Q354	S4	Q354A	No effect on the closed state.	Flynn et al. [29]
W355	S4	W355N	Destabilize the closed state (25% conductance remaining at positive voltages).	Ramentol et al. [21]
E356	S4	E356A	Destabilize the closed state (10% conductance remaining at positive voltages).	Flynn et al. [29]
N370	S5	N370W (Hydrophobic mutation)	Destabilize the closed state(25% conductance remaining at positive voltages) and destabilize the opened state. Reverse the voltage dependence when having W355-N370 double mutation.	Ramentol et al. [21]
N370	S5	N370E (Hydrophilic mutation)	Pore open and close normally.	Ramentol et al. [21]
Amino Acids	Location	Function in HCN Gating		
R367	S5	R367 and D471 interaction stabilize the closed state.		Decher et al. [32]
D471	S6			

When mutating F216, W355, and N370 individually, current conditions are detected when the membrane is depolarized. This finding suggests mutations destabilize the closed pore and cause partial opening. Thus, the author concluded that each of these three amino acids contributes to the stabilization of closed pore during depolarization [21]. Data also suggested that W355 contributes the most to closed state stabilization in the QWE motif because W355 mutation leads to the highest conductance remaining (25%) at positive voltages (Table 1). The deletion of the QWE motif in S4 also leads to immobile S4 [21,29].

Like W355N, N370W also leads to 25% conductance remaining at positive voltages. Extensive research on N370 reveals an astonishing result. First, when N370W was studied at negative voltages to simulate hyperpolarization, the current conduction decreased (partially closed channel). Thus, N370W destabilizes the closed gate during depolarization and destabilizes the opened gate during hyperpolarization [21]. Second, N370 was mutated to amino acids with different characteristics. When N370 was mutated to hydrophobic amino acids, 50% conductance remained at positive voltages and decreased conductance at extreme negative voltages. In contrast, when N370 was mutated to hydrophilic amino acids, gating is completely normal. The authors conclude that polar amino acid at 370 is required for the normal gate opening and closing [21]. Previous data has shown a water-filled crevice exposed in the cytoplasmic part of S4 when S4 moves downward at opening state [19]. The authors hypothesize that hydrophilic N370 from S5 interacting with water water-filled crevices are required to maintain the HCN channel open during hyperpolarization [21].

Finally, when the W355-N370 double mutation was created, HCN channel polarity was reversed with no change in pore structure: a hyperpolarization-activated HCN channel was converted to a depolarization-activated channel. W355N alone leads to partial gate opening during depolarization. N370W alone causes partial gate opening during depolarization and partial gate closing during hyperpolarization. However, gate polarity is reversed when both mutations present together [21]. This observation indicates that interactions created by W355(S4) and N370(S5) are working together to make the HCN channel activated on hyperpolarization. 

HCN channels play critical roles in neuropathic pain

Fundamentals of Pain

Nociception or pain is the ability to detect noxious stimuli and result from the activation of a subset of sensory neurons called nociceptors. Nociception is unique compared to other sensations because it can be activated by a wide range of external stimuli such as heat, cold, chemicals, and mechanical forces [22]. When external stimuli cause tissue damage, inflammatory factors such as prostaglandin E2 (PGE2), bradykinin, and nerve growth factors are released from damaged cells. These factors can excite the nociceptive nerve terminals to generate action potentials and enhance the pain sensation [33]. Under physiological conditions, the nociceptive system is only engaged by noxious stimuli. This type of pain is considered acute pain; it will disappear once the stimuli are removed, or the damaged tissue is repaired [22]. 

Chronic pain can be subdivided into two categories: chronic inflammatory pain and neuropathic pain. Similar to acute pain, ongoing inflammatory factors cause chronic inflammatory pain. From the traditional view, neuropathic pain is considered quite different from inflammatory pain because it can persist without stimulation or visible underlying damage [22,33,34]. Neuropathic pain is caused by direct damage to the nervous system, including metabolic disorders such as diabetes mellitus, infection such as HIV and herpes zoster, and chemotherapy. After an injury of the nervous system, the nociceptive transmission pathways exhibit hyperexcitability and produce ectopic discharges that can lead to spontaneous pain (pain without any stimuli), allodynia (pain with harmless stimuli such as light touch), and hyperalgesia (increase pain intensity with painful stimuli) [33,34].

Controversies Involved in HCN Channels' Role in Neuropathic Pain

Why does the ectopic firing of nociceptive nerves only occur in pathological conditions? Studies have provided inconsistent data, making it hard to formulate a convincing model for neuropathic pain. The inconsistency is mainly caused by two different models used to study the relationship between neuropathic pain and HCN channel: traumatic and non-traumatic models. In the traumatic model, researchers use surgical procedures (compression, resection, or ligation) to cause physical damage to large/medium-sized sensory nerves in dorsal root ganglion (DRG) [34,35]. Small-sized neurons, the majority of which are nociceptors, are focused in the non-traumatic model. Inflammation inducing agents or inflammatory states (mild thermal injury, mice with diabetes, or mice treated with chemotherapy) were used to generate neuropathic pain [23]. Because the composition of HCN isoforms is different in large somatosensory neurons and small nociceptive neurons, the channel activation mechanisms will be different. The mechanisms are so unique, suggesting there will be more than one mechanism leading to neuropathic pain based on the type of nerve injury. 

*HCN1 is the Main Contributor to Ectopic Firing in Traumatically Injured Large Neurons* 

In traumatic models, the mechanism of HCN remains mostly unclear. Using the spinal nerve ligation (SNL) model, Chaplan et al. was the first research group that provided evidence that I_h_ has a role in the hyperexcitability of the damaged large neurons in DRG. In their study, the expression of I_h_ in large-diameter DRG neurons was increased after SNL. Quantitative polymerase chain reaction (PCR) comparison of the four HCN isoforms in whole L5/6 DRGs indicates HCN1 isoform is much more abundant than other isoforms. All four HCN isoforms are found in normal and injured DRGs, with HCN1 the most abundant in both cases [35]. Another study has shown in large neurons, I_h_ was insensitive to elevations in cAMP, consistent with the expression of HCN1 [36]. Both findings suggest that HCN1 isoform is the main contributor to I_h_ production in large neurons. This idea is also supported by the fact that voltage dependence and kinetics of activation of I_h_ expressed in SNL large neurons best resemble HCN1. Additionally, Chaplan et al. almost entirely reduce tactile allodynia (pain triggered by light touch or pressure) resulting from tight ligation of the L5/6 spinal nerves with systematic administration of I_h_ blocker ZD7288. These findings demonstrate that HCN channels play an essential role in spontaneous neuronal discharge originating in the damaged large-sized neuron of DRG [35].

When I_h_ is increased after SNL, it is reasonable to speculate increasing HCN channel expression and activation. However, when Chaplan et al. studied the molecular expression of HCN isoforms, they observed marked generalized decreases in both HCN1 and HCN2 messenger RNA (mRNA) with little change in HCN3 in the ligated DRG compared with the contralateral DRG. More importantly, there is no significant difference between the activation constant of rat SNL neurons and normal human HCN1 [35]. Due to insensitive to cAMP, I_h_ current is activated by cAMP is less likely. Chaplan et al. suggest that HCN isoforms may form heteromeric complexes with non-HCN accessory proteins and accelerate their activation [35]. Other studies report that N-glycosylation and tyrosine-protein kinase regulation may influence HCN activation after nerve injury [34,37]. To our knowledge, there is no study so far that provides a detailed mechanism of HCN1in traumatically injured nerves. Their contribution to trigger and maintain neuropathic pain remains unclear, therefore requires further investigation. 

The Novel Relationship Between Neuropathic Pain and Inflammation 

It is worth mentioning that the majority of human chronic pain syndromes are due to non-traumatic causes in clinical medicine such as painful diabetic neuropathy (PDN), chemotherapy-induced peripheral neuropathy (CIPN), post-herpetic neuralgia (PHN) [33]. Thus, the traumatic model may not be the best to help us understand the pathophysiology of chronic pain. In contrast, the non-traumatic model studies focus on the function of HCN2, which is the main contributor to I_h_ in small nociceptive neurons. More importantly, the mechanism of HCN channels in the non-traumatic model is more clearly defined. A research group led by McNaughton conducted a series of studies investigating the relationship between inflammatory and neuropathic pain. Inflammatory and neuropathic pain has traditionally been regarded as discrete entities. However, McNaughton's group demonstrated that the two are closely related [14,22,23,33].

Emery et al. had several vital discoveries in the in vitro study. When deleting HCN1 in small dorsal root ganglion (DRG) neurons, most pain modalities are not affected, meaning HCN1 has no contribution to pain in small neurons, which is quite different from the traumatic model [36]. Small DRG neurons with deleted HCN2 have a significant decrease in I_h_, suggesting HCN2 is essential for generating I_h_ in small neurons. Forskolin (FSK) and PGE2, two known substances that activate adenylate cyclase, caused a membrane depolarization and a continuous firing in wild type small DRG neurons. In contrast, small DRG neurons with HCN2 deletion or wild-type neurons with nonselective HCN antagonist ZD7288 have no response to the FSK and PGE2. When using transfection to bring the HCN2 gene back to the mutated neurons, the sensitivity to FSK of the firing rate is restored [23]. We can generate a pathway from these in vitro studies: elevation of PGE2 increases the cAMP level, which activates HCN2, producing I_h_ and contributes to pain sensation. In vivo studies mainly focus on pain behavior change in mice after injecting PGE2, forskolin, or carrageenan into the hind paw. Mice with HCN2 deletion and wild-type mice treated with nonselective HCN antagonist ZD7288 are insensitive to thermal hyperalgesia [23].

Additionally, pharmacological block or genetic deletion of HCN2 does not affect the normal sensation of acute pain. Therefore, HCN2 blockage may be a useful strategy to abolish inflammatory pain without affecting normal acute pain sensation [22,33]. Emery et al. propose that inflammatory mediators, released at the nerve injury site, can promote repetitive firing of action potentials and initiate neuropathic pain [23]. This study's significance shows that the PGE2/cAMP pathway has a regulatory effect on HCN2 [33]. PGE2 is a well-known downstream product of cyclooxygenase (COX) pathways of prostanoid biosynthesis [38]. The correlation between the COX/PGE2/cAMP pathway and HCN2 implies that it is possible to treat neuropathic pain using nonsteroidal anti-inflammatory drug (NSAID) analgesic family [33].

It is widely believed that the COX/PGE2/cAMP pathway and NSAIDs are unrelated in treating neuropathic pain. However, the data above suggest that this view may need to be re-examined [23]. Several different studies support the idea of Emery et al. Research has shown that NSAIDs prevent the onset of neuropathic pain in rats [39]. When microsomal prostaglandin E synthase-1 (mPGES-1), an enzyme producing PGE2, was deleted in mice, they did not exhibit mechanical allodynia and thermal hyperalgesia over a week [40]. In diabetic mice, researchers found an increase in intracellular cAMP in nociceptive DRG neurons. The pharmacological or genetic block of HCN2 activity exerts potent analgesic effects on diabetes-associated mechanical allodynia [41]. Instead of studying the COX/PGE2 pathway directly, Resta and Mannaioni take a different approach. G Protein-Coupled Receptor 35 (GPR35) is expressed in small DRG neurons with the function of inhibiting cAMP production. Using GPR35 agonists in chemotherapy-treated mice, researchers could significantly reduce CIPN [9]. In summary, the non-traumatic model demonstrates that HCN2 has an essential role in both inflammatory and neuropathic pain because the elevated cAMP produced by the COX/PGE2 pathway is the crucial mediator for HCN2 activation. 

It is important to keep in mind that both PNS and CNS participate in neuropathic pain. It is well established that there is an increased synaptic efficacy (the ability of a presynaptic input to influence postsynaptic output) found in somatosensory neurons in the dorsal horn of the spinal cord following nerve damage. This process is called central sensitization [42]. Although both inflammatory and neuropathic pain are known to be augmented by central sensitization [43], the relationship between peripheral nerve injury and central sensitization remains controversial. Does central sensitization require continuous input from peripheral nociceptive stimulation [14]? Or once central sensitization is established, neuropathic pain can be maintained in the absence of nociceptive firing, even when the inflammation associated with peripheral nerve damage has subsided? 

Recent studies have shown that central sensitization maintenance requires continuous input from peripheral nociceptive stimulation. Three significant pieces of evidence support it. First, electrical activity present in nociceptive C-fibres of both rodents and humans even years after a nerve injury [44,45]. Second, cyclooxygenase 2 (COX2) and PGE2 levels remain elevated at the site of nerve injury for at least 18 months [39]. Third, ivabradine, the peripheral HCN blocker, is still sufficient to treat neuropathic pain in mice even long after induction of pain [14]. Thus, repetitive firing of peripheral nociceptive neurons is vital for maintaining central sensitization. Such findings imply that blocking HCN2 in peripheral nerves alone may reduce pain perception in both PNS and CNS. However, it is still unclear why COX inhibitors are ineffective in treating neuropathic pain in clinical medicine. Additional researches are required to investigate this subject. 

The analgesic effect of ivabradine still requires further research

Based on the promising results in the non-traumatic model, HCN2 can be a potential new target to treat neuropathic pain. Ivabradine is the first and only HCN channel blocker used in clinical therapy as a possible analgesic. Based on animal studies, three essential features make it a potential candidate. First, unlike ZD7288, research has shown that ivabradine does not have significant off-target effects such as acting on Na, Ca, and K ion channels [14]. Second, ivabradine cannot get into CNS because it is a substrate for P-glycoprotein (PgP), a drug transporter found in the blood-brain barrier [46]. Thus, ivabradine will not have CNS side effects like other analgesics such as opioids. Third, ivabradine did not affect acute pain thresholds, affecting normal pain behavior [14]. However, ivabradine has no selectivity to HCN isoforms [16].

Ivabradine's analgesic effect is rarely studied, most likely due to the unwanted bradycardia from HCN4 blockage. Only one research group conducted a randomized, double-blind, placebo-controlled crossover trial on HCN analgesic effect in healthy volunteers to our best of knowledge. In this study, topical capsaicin, an active component of chili peppers, is applied on forearm skin to induce an inflammatory response. Volunteers were then treated with oral ivabradine 60 minutes before cream application. Then, capsaicin cream was left on for 75 minutes and removed once the pain assessments were completed. The results are compared in the experimental and control groups, and the author concludes that ivabradine lacks analgesic effects in the capsaicin pain model [47]. A possible explanation for ivabradine's ineffectiveness in the capsaicin pain model is that the cream application is acute. Thus, the capsaicin pain model resembles acute inflammatory pain more than neuropathic pain found in patients with PDN or CIPN. Although capsaicin can increase the cAMP level, the elevation may not be long enough. Once capsaicin is removed, the cAMP level most likely starts to decrease due to metabolism. In either way, the cAMP level may not be adequate to trigger chronic pain. As mentioned previously, ivabradine does not affect acute pain [14]. The maximum acceptable dose used in the human study is 10 times lower than the ED50 for analgesia in mouse studies [47]. This may indicate dosing may not be adequate to have an analgesic effect, and local injection may provide a better outcome. Because ivabradine is a nonselective HCN blocker, bradycardia is a primary obstacle. A recent study has claimed that they discovered a new HCN2 specific drug with a completely different structure than the current HCN blockers. This study may provide a new strategy for designing and developing isoform-specific HCN channel inhibitors [48]. Thus, new drug development is the other option for future research. Few studies have shown that peripheral nerve injury can lead to blood-spinal cord barrier leakage leads to the infiltration of peripheral immune cells [49,50]. Once peripheral immune cells get into CNS, it is believed that microglia cells and astrocytes are the critical responders to inflammation and mediators in central sensitization [42]. Therefore, investigating the relationship between the HCN channels, peripheral immune cells, and cells in CNS may help us understand the molecular mechanism of the HCN channel in central sensitization and find possible new treatment options.

Limitations 

There are several limitations in our literature review. The question we are most interested in is how effective ivabradine is in treating neuropathic pain in humans. We are only able to find one research article regarding this subject. Thus, due to insufficient data, we can only briefly talk about the analgesic effect of ivabradine. The second challenge we are facing is the inability to get access to all articles. This challenge may have an impact on the quality of review on the topic of the traumatic model. We tried to find a molecular mechanism for the traumatic model but failed to do so in the available full-text articles. Those unavailable articles may have the answer to our research question. 

## Conclusions

Until today, the physiology of HCN channels is generally well understood. However, pathologies of the HCN channels that relate to neuropathic pain still require extensive research. We all know that highly conserved protein or genetic sequences often imply crucial biological functions. Ironically, this is not the case for HCN channels. Non-conserved amino acids are the keys to determine two unique features of HCN channels. The discrepancy between traumatic and non-traumatic models may suggest that different nerve injuries may cause neuropathic pain using different mechanisms. 

The process involving the HCN channel, pacemaking current, and neuropathic pain is quite complicated. There are many advanced level articles available. However, introductory-level review articles are rarely available. Our literature's unique feature is that we set our audiences as people who are entirely new to this topic, thus functioning as a stepping stone that closes the gap between experts and new learners. We believe our paper can help medical students, residents, and doctors interested in exploring new methods for pain management. There are many unexplored areas worth investigating. So far, scientists only studied physiological HCN channel structure. Are there structural differences between physiological and pathological HCN channels, which may explain its role in ectopic firing? Are there any HCN channel structural differences between the traumatic and non-traumatic nerve injury? Although we have evidence that the COX/PGE2/cAMP pathway and HCN2 are important for neuropathic pain, it is still unclear why COX inhibitors are ineffective in treating neuropathic pain in clinical medicine. The relationship between COX inhibitors and neuropathic pain should be re-examined. 
